# Shape-Dependent Optoelectronic Cell Lysis**

**DOI:** 10.1002/anie.201307751

**Published:** 2014-01-08

**Authors:** Clemens Kremer, Christian Witte, Steven L Neale, Julien Reboud, Michael P Barrett, Jonathan M Cooper

**Affiliations:** Division of Biomedical Engineering, School of Engineering, University of Glasgow, Rankine Building Oakfield Avenue, Glasgow G12 8LT (UK); Institute of Infection, Immunity & Inflammation, School of Medical, Veterinary & Life Sciences, and Wellcome Centre for Molecular Parasitology, Glasgow Biomedical Research Centre, University of Glasgow, Sir Graeme Davies Building 120 University Place, Glasgow G12 8TA (UK)

**Keywords:** cell enrichment, diagnostics, electrical cell lysis, microfluidics, optoelectronics

## Abstract

We show an electrical method to break open living cells amongst a population of different cell types, where cell selection is based upon their shape. We implement the technique on an optoelectronic platform, where light, focused onto a semiconductor surface from a video projector creates a reconfigurable pattern of electrodes. One can choose the area of cells to be lysed in real-time, from single cells to large areas, simply by redrawing the projected pattern. We show that the method, based on the “electrical shadow” that the cell casts, allows the detection of rare cell types in blood (including sleeping sickness parasites), and has the potential to enable single cell studies for advanced molecular diagnostics, as well as wider applications in analytical chemistry.

The selective lysis of cells, either to enrich samples for diagnostic assays or to target drug delivery and enhance therapy, is of considerable interest. As a consequence, a variety of techniques that result in the lysis of the cell membrane have been described, including the use of chemicals,^1^–^3^ mechanical stress,^4^–^6^ osmotic pressure,^7^, ^8^ and electrical^9^–^11^ or optical methods.^12^, ^13^ The broad applicability and speed of electrical lysis leads to it being the most widely used. Electrical lysis is initiated when the transmembrane potential (TMP) reaches a threshold, causing pores in the membrane to form and merge. The irreversible breakdown of the lipid bilayer^14^–^15^ results in unregulated transfer of ions in and out of the cell, changes in the osmotic pressures, and cell death.

Here, we describe a method (Figure 1) that enables the selective lysis of cells based upon their shape. We show how the “electrical shadow” casted by a cell onto a semiconductor surface creates a locally enhanced transmembrane field gradient, thus leading to poration and subsequent lysis. Most interestingly, the shadow is influenced by the shape of the cell, providing a method for selectively lysing different types of cells. In particular, we demonstrate that shape selectivity enables the selective lysis of small cells over larger ones, while current electrical techniques tend to favor the lysis of large cells over smaller ones, for example lysing white blood cells (WBCs) at a lower power than that required to lyse red blood cells (RBCs).^16^

**Figure 1:**
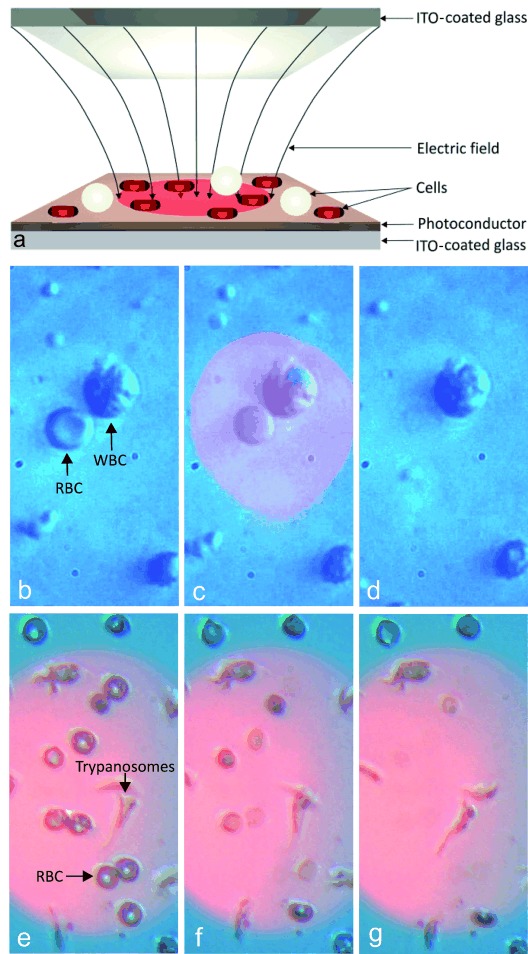
Shape-selective lysis using an optoelectronic system. a) Schematic diagram of the optoelectronic device showing the electric field concentrated in the illuminated region. b–d) A smaller RBC and a larger WBC in the illuminated region before, during, and after the light and electric field are activated (video available online, V1). The sample was suspended in a 100 mS m^−1^ buffer. c) The RBC in the illuminated region swells while the morphology of the larger WBC remains unchanged. d) 30 seconds after the voltage, 5 Vpp at 10 kHz, is turned on, the RBC has lysed and its membrane, or “ghost”, is left. e–g) A mixture of parasites and RBCs during electric-field activation with selective lysis of RBCs (video available online, V2).

To understand this process, we consider a normal healthy cell with a resting potential that depends upon the relative concentrations of cations across the membrane. Upon exposure to an electric field, this potential increases as a result of charge accumulation at the membrane.^17^ The induced TMP is not evenly distributed across the cell, as ions will accumulate in areas of highest field. A theoretical framework for this understanding was developed by Schwan,^18^ whose equation for an AC bias acting on a spherical cell in a uniform electric field showed that the frequency dependency of the applied field is of most relevance in the MHz range.^19^ Experimental observations show that above a certain voltage threshold, pores begin to form reversibly through electropermeabilisation or electroporation. If the TMP is further increased (≈1 V),^20^ the damage to the membrane is irreversible and the cell lyses. For cells placed in a uniform electric field, the voltage drop (and hence the size but not the shape of the cell) determines the differential lysis.

However, in contrast to these methods, where larger cells always lyse preferentially to smaller cells, we have developed a method that enables shape-selectivity in such a way that cells with a different geometry will preferentially lyse from within a mixture of cell types (Figure 1 b–g). To achieve this, we have developed a new approach that uses the cell itself to enhance the non-uniformity in the electric field. This technique is based on the use of a semi-conductor as one of the electrodes in the system, thus allowing cells close to this surface to affect the amount of the field within the semiconductor, and changing the electrical potential at the semiconductor liquid interface.

We also demonstrate that this technique can be implemented in a low-cost optoelectronic platform (Figure 1), where electric fields are controlled by light on an amorphous silicon film.^21^ This configuration, in which the illumination creates a virtual electrode, is already well understood, and it is known that the generated fields, which can extend over large areas, can be used to manipulate cells.^22^ This system provides us with the flexibility to study the phenomenon at the single-cell level, as well as to apply it on a larger scale without relying on complex fabrication methods.

We apply this optoelectronic technique to large-scale, electrically induced, shape-selective lysis of cells, demonstrated by selective lysis of red blood cells (RBCs) over white blood cells (WBCs) and of RBCs over trypanosomes (blood-borne parasites). This technique has the potential to facilitate downstream analysis of rare cells out of a complex population. This has significant implications for cancer diagnostics, in particular for leukemia, where the genomic and proteomic information of specific lymphocytes carries diagnostic information, while the molecular profiles of circulating tumor cells has been linked to prognosis^23^ and the efficiency of therapies.^24^

Figure 1 a shows a schematic of the system, where human RBCs are placed onto an amorphous silicon chip, and the cells in the illuminated region are exposed to a larger electric bias. As expected, in this configuration, the RBCs will lie flat on the surface of the semiconductor electrode, thus resulting in their shape having a larger impact on the drop of the potential than the shape of the WBCs. The subsequent process of cell lysis involves poration and swelling, with the eventual loss of contrast (Figure 1 a–c). Similarly, we exploit the geometrical difference between trypanosomes and RBCs to specifically lyse RBCs (Figure 1 e–g).

To optimize the efficiency of the method, we modelled the system (COMSOL Multiphysics 3.5), where cells were represented by a single-shelled model with a 7 nm membrane, using a 2D geometric cross section through the cells and electrodes (Figure 2 a). The values used are given in Table T1 in the Supporting Information. The conductivity of the amorphous silicon was represented by a saturated Gaussian profile.^25^ This represents the effect of the light pattern that is created by focusing the light from a data projector (Dell 1510X) through an upright microscope (Olympus BX51), so that the maximum of the saturated Gaussian profile corresponds to the illuminated areas (light state). WBCs were modelled as being circular (10 μm radius) while RBCs were considered biconcave (3 μm height, 8 μm width). Cells were modelled in close proximity to the chip, with a 100 nm gap (see theory section in the Supporting Information, and references [1] and [2] therein).

**Figure 2:**
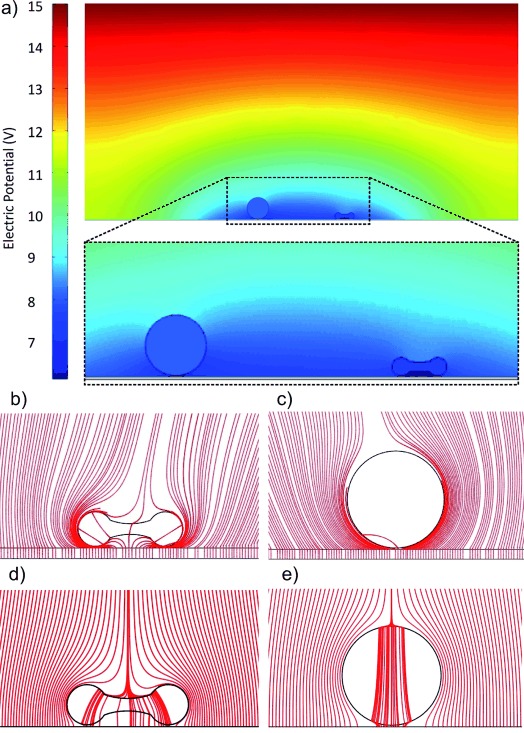
The electrical shadow. a) WBC (sphere) and RBC (biconcave disk) on a 1 μm layer of amorphous silicon, surface plot depicts potential (V), the height and width of the cross section are 100 μm and 200 μm, respectively. Media conductivity was 100 mS m^−1^, voltage 15 Vpp at 10 kHz. The inset is a detail of the field around the cells, showing the low field under the RBC. b–e) Close-up view of the field lines around individual cells, showing how the cells cause the field lines to deviate around them when placed on amorphous silicon (b: RBC, c: WBC), this effect is dramatically decreased when the cells are located on a gold surface (d: RBC, e: WBC).

Figure 2 b,c show the field lines deflected by both the RBC and WBC. The model demonstrates that the cell itself affects the potential beneath it (literally, the cell casts an electrical shadow, which is represented as a darker blue, lower-potential region in Figure 2 a). The extent of this shadow depends on the distance between the cell and the illuminated semiconductor. If the cells are positioned at a greater distance from the semiconductor, the model shows that the shadow that is cast is less pronounced (Figure S6 in the Supporting Information), a fact that is supported by experimental data, which show that the cells do not lyse (Figure S9 in the Supporting Information).

The model also shows that the shape of the electrical shadow correlates to the geometry of the cell. The relatively wide and flat biconcave RBCs cause a greater deflection of the field lines around them. This leads to a concentration of these field lines at their edges and underneath them, resulting in large electrical fields in these locations. The spherical WBCs also increase the strength of the electric field around them, but dramatically less so than the RBCs, thus providing a handle to lyse cells specifically depending on their shape.

Furthermore, we used the model to investigate whether this same phenomenon could be observed with a good electrical conductor, such as gold or platinum, traditionally used as an electrode in cell lysis experiments. Metals, as good electrical conductors, will have a constant potential across their surface, and, as expected, this reduces the ability of the cell to change the electrical fields around it (Figure 2 d,e). This correlates with our experimental observations that, while it is possible to lyse cells with metal electrodes, shape-selective lysis is not possible. Using our model, we calculated the induced TMP across the membranes of the cells for different parameters (Figure 3 a). The voltage drop was determined through the center of the cell (Figures S1 and S6 in the Supporting Information). The model predicts that the TMP of RBCs will be greater than 1 V at 10 kHz, but will drop to less than 0.4 V at 70 kHz. While the TMP of the WBC is higher at higher frequencies, it can be seen that it is smaller than that of the RBC if the applied AC bias is below 30 kHz. This provides the basis for the selective lysis of the RBCs over the WBCs.

**Figure 3:**
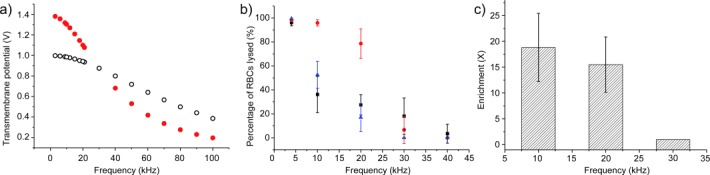
TMP and lysis efficiency as a function of the frequency. a) TMP obtained from simulations. The simulated cell’s induced TMP across the membrane (V) for different frequencies (kHz) for RBCs (red dots) and WBCs (circles). At low frequencies, the RBCs have a larger TMP, allowing them to be lysed while leaving the WBCs intact. b) Effect of frequency and medium conductivity on the percentage of lysed RBCs (medium conductivities: 25 mS m^−1^ black squares, 100 mS m^−1^ red circles, 1400 mS m^−1^ blue triangles). c) Enrichment of WBCs over RBCs. Ratio of the number of WBCs over the number of RBCs after lysis, compared to the original ratio (medium conductivity of 100 mS m^−1^). Error bars are the standard deviation over six experiments.

These events occur in media with a range of conductivities from 25 mS m^−1^ to those of PBS at approximately 1400 mS m^−1^. However results in Figure 3 b show that there is an optimum conductivity at 100 mS m^−1^, for which lysis efficiency is above 75 % across a wide range of frequencies.

Importantly, this process of selective lysis was observed in high-conductivity media, negating the need for sample dilution prior to enrichment (for frequencies <20 kHz, increasing numbers of the larger cells were lysed). In one example, using optimized conditions of 100 mS m^−1^ buffer conductivity and a frequency of 20 kHz, we demonstrated a circa twenty-fold enrichment of WBC with respect to RBCs (Figure 3 c). At the frequency of 30 kHz, corresponding to the cut-off frequency around which the selectivity for RBCs over WBCs is lost (Figure 3 a), there was no significant enrichment of WBCs.

While the observation of the lysis of a few cells under high magnification is important to understand this new technique, one benefit will come from the lysis of a large number of cells of one type while leaving another intact. Here we show shape-selective lysis of RBCs over trypanosomes (motile unicellular parasitic protozoa, some species of which are responsible for conditions including African sleeping sickness and Chagas’ disease^26^). Trypanosomes have a similar thickness (3 μm) to RBCs but are more elongated (20 μm in length). Selective lysis is shown over a period of 4 s at 14 Vpp and 3 kHz in Figure 1 d–f. Figure 4 shows similar, lower-magnification experiments, demonstrating that the technique can be used to enable the enrichment and the identification of rare cells in larger sample volumes.

**Figure 4:**
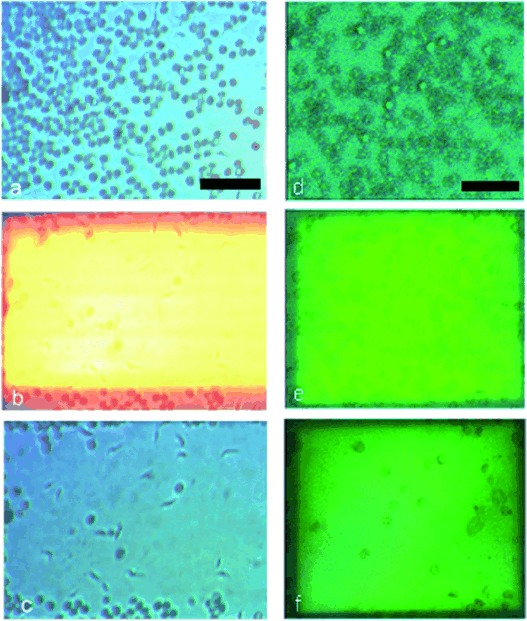
Selective lysis of RBCs on a larger scale viewed with a 10× objective over trypanosomes and WBCs at low magnification in a buffer with a conductivity of 100 mS m^−1^. a) Sample of RBCs and trypanosomes before lysis (240 RBCs and 19 trypanosomes present in a sample of whole blood diluted 20 times in the buffer); b) during lysis, (12 s, 10 Vpp, 10 kHz); c) after lysis, where only 10 RBCs remain while all the trypanosomes are intact, showing the power of this technique to allow the identification of the distinct shapes of the trypanosomes in the sample after the RBCs have been lysed, which were not obvious before. d) A sample of RBCs and WBCs before lysis in a ratio of 19:1. e) During lysis (30 s, 15 Vpp, 10 kHz), and f) after lysis, where the ratio is 1:1, resulting in the enrichment of the number of WBCs, evident as larger cells, over the RBCs, which preferentially lyse at these conditions, with hundreds of RBCs lysing in this one experiment. Scale bar is 50 μm. The difference in the appearance in these two experiments is due to the light that is used to illuminate the optoelectronic device being more heavily filtered out in d–f to allow the imaging of the lysis process as it happens.

In these large-area processing experiments, the cells were spread so that they form a single layer close to the surface as overlapping or “stacks” of cells shield each other, changing local TMPs. A roadmap calling for elimination of the trypanosomiases by 2020^27^ will require improved diagnosis of these diseases, and the methodology set out here offers potential toward detecting parasites in low-parasitaemia infections.

Previous work, reported for optoelectronic devices^28^, ^29^ has shown that a higher electrical field in the illuminated region leads to a sufficient increase in TMP to induce lysis. Our work now demonstrates how, with a deeper understanding of the process involved, one can adapt the system to enable shape-selective cell lysis.

The technique also has the potential to be integrated with dielectrophoretic manipulation of living cells, the traditional application of these optoelectronic platforms,^30^ where the projection of the cell onto the surface, and consequently its electrical shadow, can be controlled through the dielectrophoresis force.^25^ The natural integration of both techniques has the potential to selectively lyse any combination of cell types.

In conclusion, we have shown a method whereby the shape, not size, of the cell determines the electrical conditions that result in lysis. We demonstrated that the technique can be performed over large areas, which could be several mm^2^. This provides the potential to process many hundreds or thousands of cells simultaneously and could therefore be an initial sample-processing step for a diagnostic application. To illustrate this potential, we show a 20-fold enrichment of white over red blood cells. Indeed, one appealing aspect of the technology is its low cost, another the flexibility in its use (where one can choose the area of cells to be lysed simply by redrawing the pattern). In the future, the method may be useful in detecting other rare cell types in blood, including metastasing cancer cells.

## Supporting Information

As a service to our authors and readers, this journal provides supporting information supplied by the authors. Such materials are peer reviewed and may be re-organized for online delivery, but are not copy-edited or typeset. Technical support issues arising from supporting information (other than missing files) should be addressed to the authors.

miscellaneous_information

v1

v2
